# Feasibility Evaluation of Partially Replacing Ordinary Portland Cement with Ferro-Nickel Slag in Ready-Mixed Concrete for Precast Applications

**DOI:** 10.3390/ma18184315

**Published:** 2025-09-15

**Authors:** Jin-Su Kim, Jun-Pil Hwang, Chang-Hong Lee, Jang-Ho Jay Kim

**Affiliations:** 1 School of Civil and Environmental Engineering, Yonsei University, 50, Yonsei-ro, Seodaemun-gu, Seoul 03722, Republic of Korea; kjinsu@yonsei.ac.kr; 2 Concrete Materials Mechanics Engineering (CMME) Co., Ltd., 11, Tojeong-ro 35-gil, Mapo-gu, Seoul 04166, Republic of Korea; yellowjp@naver.com; 3 Posco E & C Co., Ltd., 11, Incheon tower-daero, Yeonsu-gu, Incheon 21982, Republic of Korea; changhong@poscoenc.com

**Keywords:** eco-friendly, industrial waste, ferro-nickel slag, steam curing, precast

## Abstract

The global generation of industrial waste is increasing rapidly, with much of it either landfilled or discharged into marine environments, resulting in severe environmental pollution. To address this issue, extensive research has been conducted on utilizing waste materials as partial replacements for cement. Although concrete incorporating industrial by-products offers environmental advantages—such as reducing pollution and lowering CO_2_ emissions—its application has been limited by poor early-age performance. In South Korea, the annual production of ferronickel slag (FNS) now exceeds 2,000,000 tons, yet its usage remains minimal. To improve this early-age performance, researchers have applied steam curing (SC), a method widely used in precast concrete, which can enhance the utilization of FNS-containing concrete. Some studies have individually evaluated the mechanical or microstructural properties of SC effects, but the combined effects of FNS and SC replacement in precast concrete have rarely been addressed. This study applied SC, a method widely used in precast concrete production, to improve the performance of FNS concrete and conducted a comprehensive evaluation to promote its practical application. For this purpose, ordinary Portland cement (OPC) was partially replaced with FNS at rates of 10%, 20%, and 30%. To assess the effects, tests were conducted on hydration heat, SEM, and XRD, along with evaluations of compressive and splitting tensile strength. Results identified 20% as the optimal replacement ratio. At this ratio, chloride penetration resistance and freeze–thaw durability were also assessed. Furthermore, FNS concrete was evaluated under both natural curing (NC, 28 days) and SC conditions to simulate precast production. Under NC, mechanical properties declined as the FNS content increased, whereas under SC, the performance of the 20% replacement mixture was comparable to that of the control. In addition, the chloride diffusion coefficient and freeze–thaw resistance were improved by 11% and 2%, respectively, under SC compared to NC. This study evaluated the feasibility of FNS-containing concrete, and further studies should be conducted to investigate the structural performance of FNS-containing reinforced concrete via methods such as flexural, shear, splicing, and debonding experiments.

## 1. Introduction

The global generation of industrial waste has been increasing rapidly. This waste is either landfilled or discharged into marine environments, leading to severe environmental pollution. To address industrial waste management and environmental pollution, extensive research has been conducted, particularly on the use of industrial waste as recycled materials to replace cement [1,2,3]. Many research institutions are investigating recycled industrial by-products—including ground granulated blast furnace slag (GGBFS), silica fume, fly ash, and ferronickel slag (FNS)—as alternative construction materials. When used as admixtures, these materials can contribute to the production of more cost-effective and sustainable concrete [4,5,6].

Concrete incorporating industrial by-products enhances environmental sustainability by significantly reducing CO_2_ emissions during production. As shown in Table 1, cement substitutes reduce CO_2_ emissions at varying rates. Crossin et al. reported that replacing cement with GGBFS, silica fume, fly ash, and FNS reduced CO_2_ emissions by 47%, 8–12%, 22–30%, and up to 77%, respectively [7,8,9,10]. These values, based on laboratory experiments and life-cycle assessments, demonstrate the environmental potential of supplementary cementitious materials (SCMs). However, the use of recycled industrial materials as cement substitutes has been limited due to their low early-age performance. In South Korea, the annual production of FNS exceeds 2,000,000 tons, yet its utilization remains minimal, with the majority being landfilled [11]. To promote the effective use of FNS, it is essential to improve the performance of FNS-based concrete. According to Ahmad et. al., the early-age compressive strength of GGBFS, fly ash, and FNS was 60–80%, 55–80%, and 60–70%, respectively [5,12,13].

Steam curing (SC) has been shown to accelerate cement hydration and enhance the quality of concrete [14,15]. Ramezanianpour et al. reported that increasing the SC temperature in precast concrete accelerates hydration, promotes the formation of calcium–silicate–hydrate (C-S-H), and improves the compressive strength [6,15,16,17]. Their study found that the early-age compressive strength relative to 28-day strength was 39%, 46%, and 53% at maximum temperatures of 50 °C, 60 °C, and 70 °C, respectively, indicating that higher SC temperatures promote faster strength development. Vahid et al. [18,19,20] further observed that SC reduces air permeability and chloride diffusion in precast concrete. In addition, SC is widely adopted in the construction industry due to its practicality and compatibility with precast concrete. Therefore, a systematic investigation of the effects of SC on FNS concrete is necessary to enhance its performance. Edwin et al. evaluated the compressive strength of concrete incorporating various FNS replacement ratios and compared the results obtained under natural curing (NC) and SC [17]. Their results showed that SC improved compressive strength. Similarly, Baoliang et al. reported that applying SC to FNS concrete promoted the formation of calcium–aluminum–silicate–hydrate (C-A-S-H), thereby enhancing resistance to sulfate attack [20]. However, research on the combined effects of SC and FNS on concrete performance remains limited. Previous studies on FNS have focused on hydration properties (e.g., XRD and SEM analyses) or specific durability characteristics (e.g., sulfate resistance). Furthermore, some studies have individually evaluated the mechanical or microstructural properties of SC effects, but the combined effects of FNS and SC replacement in precast concrete have rarely been addressed. This study compares the mechanical properties of concrete made with 100% ordinary Portland cement (OPC) with those of concrete in which 10%, 20%, and 30% of OPC were replaced with FNS. To conduct a comprehensive evaluation of the impact of SC on FNS concrete, microstructural characteristics, compressive strength, split tensile strength, and durability were evaluated. Hydration behavior was analyzed using hydration heat measurements, scanning electron microscopy (SEM), and X-ray diffraction (XRD). The optimal FNS replacement ratio was determined based on the mechanical strength results. In addition, chloride penetration and freeze–thaw resistance were assessed for the mixture with the optimal FNS content to evaluate its practical applicability. This study evaluated the feasibility of FNS-containing concrete, and further studies should be conducted to investigate the structural performance of FNS-containing reinforced concrete via methods such as flexural, shear, splicing, and debonding experiments.

## 2. Mix Proportion and Specimen Details

### 2.1. FNS Mixing and Curing Proportions

In this study, FNS was pulverized as finely as cements to manufacture the specimens for the evaluation of strength characteristics, and washed aggregates were purchased to ensure the absence of impurities in the aggregates. OPC and FNS were used as binders with a water-to-binder ratio of 40%. The maximum size of the coarse aggregate, the fine aggregate ratio, the air content, and the slump were 25 mm, 47%, 5%, and 100 mm, respectively. The target compressive strength after 28 days was 35 MPa. Table 2 shows the mix proportions used in this study. To evaluate the effect of FNS on concrete properties, OPC was partially replaced with FNS at replacement ratios of 10%, 20%, and 30%. To achieve performance comparable to concrete mixed with OPC of 100%, a high replacement ratio cannot be used. Three specimens were cast for each of the compressive strength and split tensile strength tests. For NC, the specimens were cured at a temperature of 23 °C and relative humidity of 65% according to ASTM C192 [21]. For SC, the specimens were cured in an oven at a temperature of 90 °C and a relative humidity of 95% for 2 days. According to Lee et al., more than 90% of cement hydration occurs when concrete is cured for two days at temperatures exceeding 90 °C [22,23,24]. The heating of the SC process was set at a rate of 10 °C/min and maintained at the target temperature of 90 °C for 2 days. The cooling was maintained at laboratory temperature (20 °C) for more than 8 h.

### 2.2. Substrate Materials

This study involved the partial replacement of cement with FNS, making a comparison of their chemical compositions essential for understanding potential impacts on concrete properties. The average particle size, specific surface area, and loss on ignition (LOI) of FNS were 3.39 µm, 4666 cm^2^/g, and 0.01%, respectively. Table 3 shows the chemical compositions of OPC and FNS. The CaO, MgO, SiO_2_, and Fe_2_O_3_ contents in OPC were 60.99%, 3.14%, 20.47%, and 3.27%, respectively. In contrast, FNS contained 6.60% CaO, 43.40% MgO, 40.70% SiO_2_, and 7.90% Fe_2_O_3_. Compared with OPC, FNS exhibited a significantly lower CaO content and higher levels of SiO_2_, Fe_2_O_3_, and MgO. The significant differences in CaO and MgO contents were considered critical factors influencing the behavior of FNS in concrete.

The chemical compositions were analyzed using powdered specimens obtained from the control and the FNS 10%, FNS 20%, and FNS 30%. Table 4 shows the oxide content of each blend. Among the components evaluated, CaO and MgO showed the most significant changes with increasing FNS replacement ratios. As the FNS content increased, the CaO content decreased by a factor of 1.25–1.37 compared to the control specimen, whereas the MgO content increased by a factor of 3–5. These trends in CaO reduction and MgO increase influence the mechanical strength of concrete. The compound formation and reactivity observed in XRD and SEM analyses further support these findings.

## 3. Hydration and Microstructural Characterization

### 3.1. Hydration Heat Under Various FNS Replacement Ratios

The heat of hydration in concrete can be measured using three primary methods: (1) adiabatic temperature rise testing, (2) semi-adiabatic temperature rise testing, and (3) isothermal calorimetry. The adiabatic temperature rise test evaluates the heat generated under conditions where no heat is lost to the surroundings. The concrete specimen is insulated in this method to prevent thermal dissipation during the hydration process. The heat of hydration was assessed using the adiabatic temperature rise test, which minimizes external thermal influences and allows for thermal correction, in accordance with ASTM C186 [25]. The test duration was set to 14 days from the time of concrete casting. The hydration characteristics of each specimen were compared and analyzed using raw and after-correction data. The results were then applied to the heat of hydration estimation, Equation (1), which predicts the adiabatic temperature at any curing time t:

(1) $$T=K(1-e^{-\alpha t})$$

where T is the adiabatic temperature rise at time t [°C], K is the maximum temperature rise [°C], α is the reaction rate, and t is the time [days].

**Figure 1:**
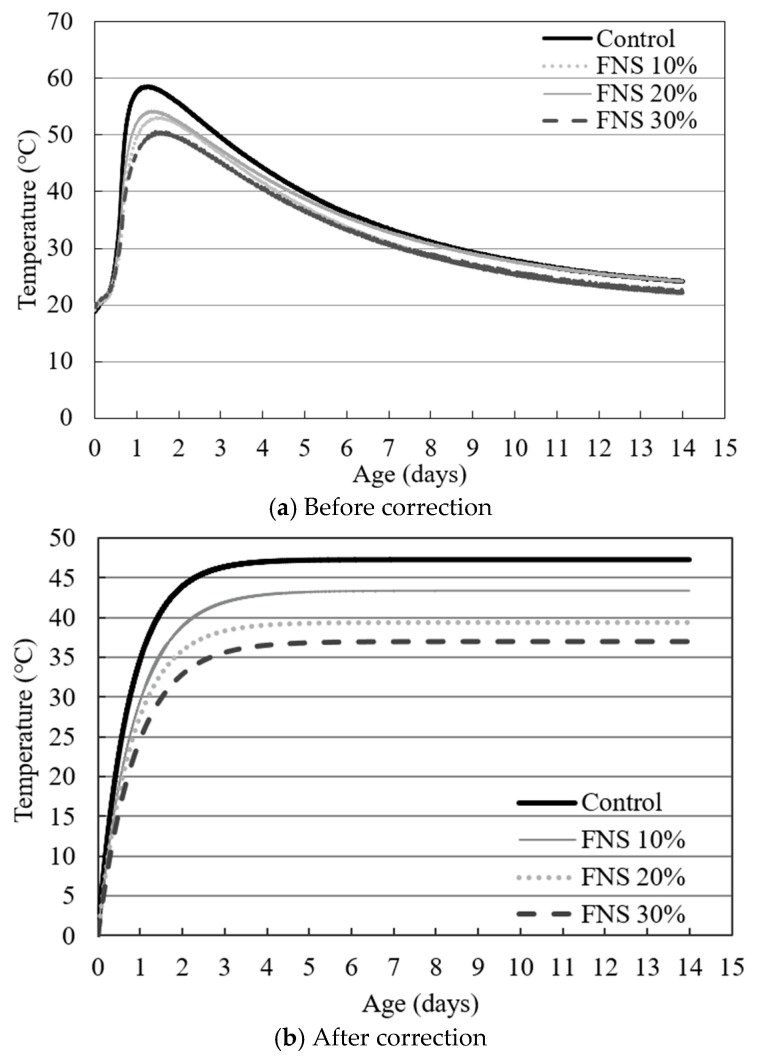
Results of the adiabatic temperature rise test.

Figure 1a and Table 5 show the results of the adiabatic temperature and the fastest reaction rate. The control specimen exhibited the highest maximum temperature increase (K) and the fastest reaction rate (α). As the FNS replacement ratio increased, both the temperature rise and reaction rate decreased. Figure 1b shows the results after correction. All specimens reached peak temperature within 1–2 days and then gradually declined over the 14-day curing period. At 2 days, the adiabatic temperatures for the control, FNS 10%, FNS 20%, and FNS 30% were 43.92 °C, 38.91 °C, 35.84 °C, and 32.86 °C, respectively. At 14 days, the corresponding temperatures were 47.3 °C, 43.4 °C, 39.4 °C, and 37.0 °C. The fastest reaction rate revealed 1.3220, 1.2050, 1.1370, and 1.0980 for the control, FNS 10%, FNS 20%, and FNS 30%, respectively. FNS is composed of SiO_2_, MgO, CaO, Al_2_O_3_, and Fe_2_O_3_, with the amorphous minerals typically exceeding 50%. According to Huang, the incorporation of FNS powder can delay the early hydration of concrete, mainly due to its chemical composition and the presence of amorphous and Mg-rich phases [12,26,27].

### 3.2. Comparison of SEM Under Various FNS Replacement Ratios and Curing Methods

**Figure 2:**
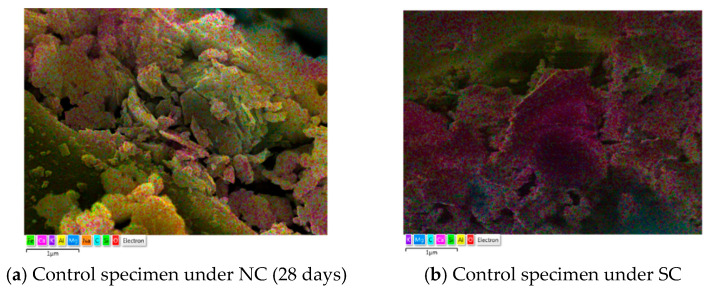

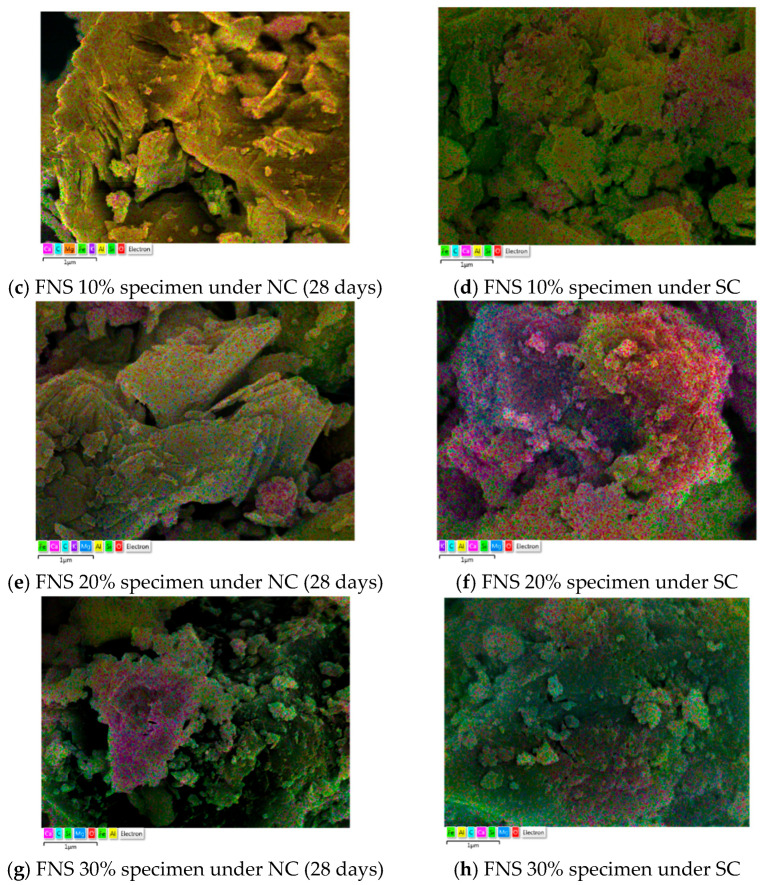
SEM-EDS analysis of specimens cured using different methods with varying FNS replacement ratios.

SEM involves directing an electron beam onto a sample to produce high-magnification images of its surface. When electrons interact with the sample, signals such as secondary electrons, backscattered electrons, transmitted electrons, and X-rays are generated and captured for imaging purposes. This process, conducted under vacuum conditions, allows detailed observation of the sample’s surface morphology. Additionally, qualitative and quantitative elemental analyses can be performed using energy-dispersive spectroscopy (EDS), which is based on the intensity of energy emitted from the sample [28]. SEM-EDS analysis was conducted under both the NC (28 days) and SC conditions. The results are shown in Table 6 and Figure 2. Under NC, the SEM images of the control specimen showed a dense microstructure with uniformly distributed gel-like hydration products. In contrast, specimens containing FNS exhibited increased porosity and greater microstructural heterogeneity as the replacement ratio increased. Under SC, hydrated gels appeared more agglomerated, and overall microstructural density improved compared to NC. Notably, the FNS 30% specimen under SC exhibited a uniform distribution of gel phases between crystalline particles, in contrast to the less compact structure observed under NC.

According to the EDS analysis, the Ca content (wt%) in the control specimen under NC was 3.60%, decreasing to 2.01% with a 20% FNS replacement ratio due to the low CaO content in FNS. The Mg content increased with higher FNS replacement ratios, recorded as 0.59%, 2.38%, 13.13%, and 14.44% for the control, FNS 10%, FNS 20%, and FNS 30%, respectively. The Si content remained relatively stable across all mixes, within the range of 10–15%, regardless of the replacement ratio. In contrast, the Mg content increased consistently with higher FNS replacement ratios, recorded as 0.59%, 2.38%, 13.13%, and 14.44% for the control, 10% FNS, 20% FNS, and 30% FNS specimens, respectively. The Fe content was also higher in all FNS specimens compared to the control. A comparison of the EDS results for the FNS 30% specimens under NC and SC revealed that all elemental contents, except for Si, decreased under SC. Specifically, the Ca, O, Mg, and Fe contents under SC were 7.46%, 46.62%, 9.13%, and 3.95%, respectively. These findings confirm that both the curing conditions and the FNS replacement ratios have a significant influence on the microstructure and elemental composition of concrete. In particular, SC enhances the agglomeration and density of hydration products.

### 3.3. Comparison of XRD Under Various FNS Replacement Ratios and Curing Methods

**Figure 3:**
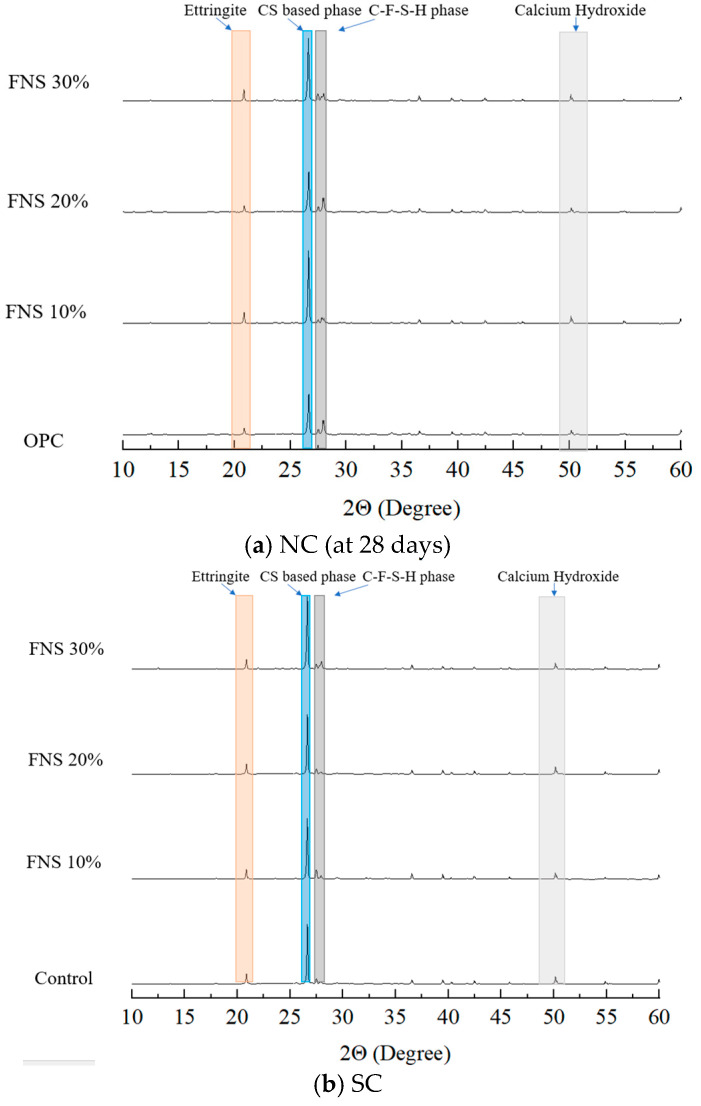
XRD patterns of specimens cured under different methods with varying FNS replacement ratios.

XRD is a technique that involves irradiating crystalline materials, characterized by regular atomic arrangement with X-rays. When X-rays interact with electrons in the crystal lattice, they produce secondary spherical waves through a phenomenon known as elastic scattering. The regular and precise scattering pattern generates a diffraction profile, which enables the identification of the elemental and mineralogical composition of the material [29]. The XRD results are shown in Table 7 and Figure 3.

The major phases identified across the specimens included ettringite (Ca_6_(Al(OH)_6_)·2(SO_4_)_3_·26H_2_O), CS-based phase (C-S-H, 3CaO·SiO_2_, 2CaO·SiO_2_), C-F-S-H phase, and calcium hydroxide (Ca(OH)_2_). Under NC, the intensity of CS-based phases (26.60°) was 56,869 for the control specimen and increased to 73,276 with FNS 30%. Under SC, the corresponding CS-based phase intensities were 57,503 for the control and 70,155 for the FNS 30%. Under NC, the intensity of the CS phase (26.60°) was 56,869 for the control specimen and increased to 56,860, 65,675, and 73,276, respectively, with 10%, 20%, and 30% FNS replacement ratios. Under SC, the corresponding CS-based phase intensities were 57,503 for the control and 58,053, 57,578, and 70,155 for the 10%, 20%, and 30% FNS specimens, respectively. These results demonstrate a general increase in CS-based phase intensity with higher FNS content under both curing methods. Consistent with the SEM-EDS findings, the proportion of CS-based phase under SC was slightly lower than that under NC.

For the C-F-S-H phase, under NC, the intensity values were 4647 for the control and 7115, 5255, and 3822 for the FNS 10%, FNS 20%, and FNS 30%, respectively. Under SC, the C-F-S-H phase’s intensities were 3908 for the control and 3863, 2169, and 7602 for the FNS 10%, FNS 20%, and FNS 30%, respectively, showing no clear pattern across replacement levels. The C-F-S-H phase is a secondary hydration product formed by the reaction of Fe- and Si-containing phases. Its formation is influenced by several factors, including the FNS replacement ratio, the solubility of Fe and Si ions, and the curing conditions. Variations in peak intensity result from the low crystallinity of the hydration products and overlapping signals, which make the quantification of the C-F-S-H phase difficult.

Calcium hydroxide intensity generally increased under SC, particularly from 6930 to 9232 at the FNS 10%, indicating enhanced hydration. This indicates generally higher calcium hydroxide under SC, particularly in specimens with lower FNS content. CS-based phase and calcium hydroxide were generally higher under SC for the control, FNS 10%, and FNS 20%, indicating that SC can produce hydration effects comparable to 28-day NC, supporting its applicability in precast concrete.

Comparative XRD analysis with the SEM-EDS results showed that the microstructures of specimens containing FNS had increased porosity and heterogeneous gel distribution compared to the control specimen. In addition, the SEM-EDS results showed that the SC specimens had a microstructure with a denser and more uniform distribution of hydration products than the NC specimens. This suggests that the densification of the microstructure caused by SC could be revealed by the formation of CS-based phase and calcium hydroxide, which provide a microstructure favorable for strength development.

## 4. Mechanical Property and Durability

### 4.1. Comparison of Compressive Strength Under Various FNS Replacement Ratios and Curing Methods

**Figure 4:**
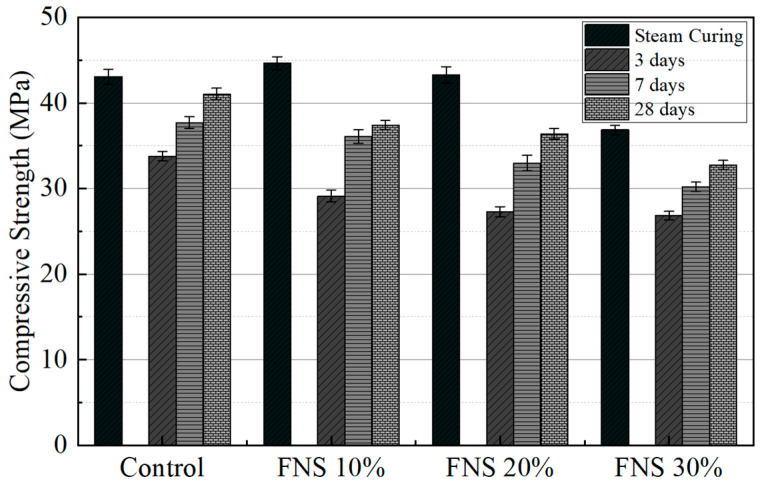
Compressive strength of specimens under different curing methods with varying FNS replacement ratios.

Cylindrical concrete specimens (Φ100 × 200 mm) were prepared according to the ASTM C192. Compressive strength was measured at 3, 7, and 28 days of curing to evaluate early-age, intermediate, and standard-age strength, respectively, in accordance with ASTM C39 [30]. At 28 days, the target compressive strength was 35 MPa. Loading was applied at a rate of 0.6 MPa/s using a universal testing machine with a maximum load capacity of 1000 kN and a measurement accuracy of 500 N. Compressive strength tests were conducted under both NC (3, 7, and 28 days) and SC, and the results are shown in Figure 4. Under NC, a general decrease in compressive strength was observed as the FNS replacement ratio increased, with a particularly noticeable reduction at early ages (3 and 7 days). At 3 days, the compressive strength of the control specimen was 33.79 MPa, whereas the strengths of FNS 10%, FNS 20%, and FNS 30% were 29.13 MPa, 27.27 MPa, and 26.83 MPa, respectively. At 7 days, the control specimen reached 37.69 MPa, while FNS 10%, FNS 20%, and FNS 30% achieved 36.08 MPa, 32.98 MPa, and 30.22 MPa, respectively. At 28 days, the compressive strengths were 41.07 MPa (control), 37.43 MPa (FNS 10%), 36.40 MPa (FNS 20%), and 32.75 MPa (FNS 30%), indicating a gradual reduction in strength with increasing FNS content. The controls, FNS 10%, and FNS 20% all exceeded the target strength of 35 MPa, whereas the FNS 30% did not achieve the target strength. The correlation in adiabatic temperature revealed compressive strength losses of −14%, −19%, and −21% for FNS 10%, FNS 20%, and FNS 30%, respectively. Similarly, the decrease in the fastest response parameter (α) was consistent with the reduced 7-day strength development. At day 28, the strength losses narrow partially, but the trend remains consistent.

Under SC, the compressive strengths were generally higher than those under NC at 28 days, particularly for FNS 10% and FNS 20%. The compressive strengths of the SC specimens were 44.64 MPa (FNS 10%) and 43.28 MPa (FNS 20%), representing increases of more than 20% compared to their respective NC values [6,15,16,17]. In contrast, the SC specimen with 30% FNS achieved 36.85 MPa, which was lower than the control specimen at 43.07 MPa. The highest compressive strength was observed in the SC mix with FNS 10%, followed closely by the FNS 20%, both of which showed comparable or superior performance relative to the control specimen. In contrast, the 30% FNS mix exhibited reduced strength under both curing conditions. These results suggest that the effectiveness of SC in improving strength diminishes when the FNS replacement ratio exceeds a certain threshold. Therefore, a replacement level of 20% is considered optimal, as it balances compressive strength performance and resource recycling efficiency. In addition to its mechanical benefits, SC compensates for the early hydration delay induced by FNS, promotes microstructural densification, and enhances the uniform distribution of hydration products, thereby facilitating the development of strength.

### 4.2. Comparison of the Split Tensile Strength Under Various FNS Replacement Ratios and Curing Methods

**Figure 5:**
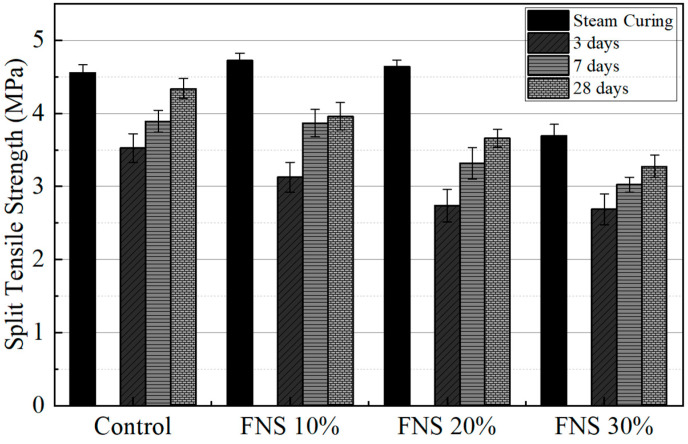
Split tensile strength of specimens under different curing methods with varying FNS replacement ratios.

Cylindrical concrete specimens (Φ100 × 200 mm) were prepared according to ASTM C192, and split tensile strength was evaluated following ASTM C496 [31]. Tests were conducted under both NC (3, 7, and 28 days) and SC, with results shown in Figure 5. Overall, the split tensile strength exhibited a trend similar to that of the compressive strength. Under NC, the split tensile strength at 3 days was 3.52 MPa for the control specimen, and 3.12 MPa, 2.74 MPa, and 2.69 MPa for FNS 10%, FNS 20%, and FNS 30%, respectively, indicating a decrease in strength with increasing FNS content. At 7 days, the values were 3.89 MPa (control), 3.87 MPa (FNS 10%), 3.32 MPa (FNS 20%), and 3.02 MPa (FNS 30%). At 28 days, the strength was 4.34 MPa (control), 3.96 MPa (FNS 10%), 3.66 MPa (FNS 20%), and 3.28 MPa (FNS 30%). Notably, the FNS 10% mix demonstrated a split tensile strength comparable to that of the control specimen at 28 days, whereas the FNS 30% mix consistently recorded the lowest strength at all curing ages. The correlation in adiabatic temperature revealed splitting tensile strength losses of −11%, −22%, and −24% for FNS 10%, FNS 20%, and FNS 30%, respectively. The trend of split tensile strength losses was similar to compressive strength losses.

Under SC, the split tensile strength of the control specimen was 4.56 MPa, whereas the strengths of the FNS 10%, FNS 20%, and FNS 30% were 4.72 MPa, 4.64 MPa, and 3.69 MPa, respectively. Both the FNS 10% and FNS 20% showed higher strength than the control specimen, indicating a positive effect on FNS replacement under SC conditions. These results suggest that when combined with SC FNS replacement, up to 20% of the split tensile strength can be maintained or enhanced, offering environmental and resource-saving benefits without compromising mechanical performance.

### 4.3. Comparison of Chloride Penetration Resistance Under the Curing Methods

**Figure 6:**
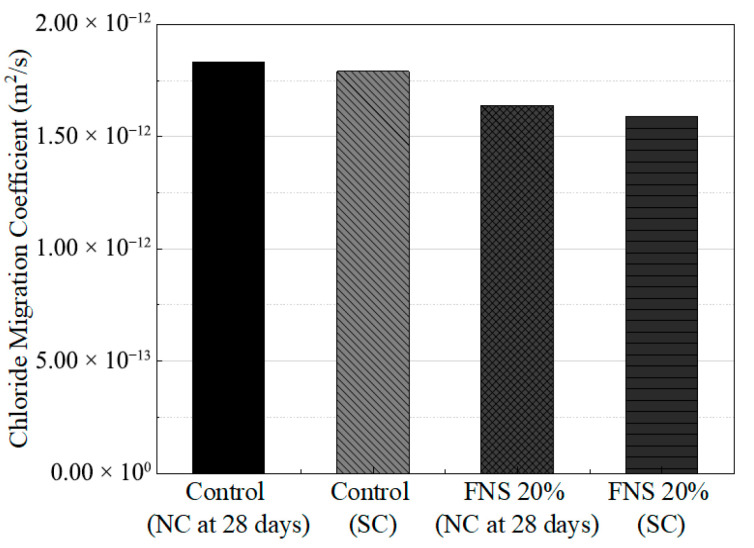
Chloride migration coefficients of specimens under different curing methods at the optimal FNS replacement ratio.

The chloride ion penetration resistance test was conducted in accordance with the NT-BUILD 492 standard. The control specimen and the optimal mix design incorporating FNS 20% were compared. Cylindrical concrete specimens (Φ100 mm × 50 mm) were first saturated in a Ca(OH)_2_ solution under vacuum for 1 day to fill internal pores [32]. Detailed calculation formulas are provided in Appendix A.1. Figure 6 shows the evaluation results. Under NC (28 days), the chloride diffusion coefficients for the control and FNS 20% specimens were measured as 1.83 × 10^−12^ m^2^/s and 1.64 × 10^−12^ m^2^/s, respectively. Under SC, the corresponding values were 1.79 × 10^−12^ m^2^/s (control) and 1.59 × 10^−12^ m^2^/s (FNS 20%). In all cases, the FNS 20% exhibited lower chloride diffusion coefficients than the control. Furthermore, SC specimens showed improved resistance relative to those under NC. These results indicate that FNS 20% not only maintained or improved mechanical properties such as compressive and tensile strength but also demonstrated superior durability in resisting chloride ingress. Notably, the FNS 20% specimen under SC exhibited a diffusion coefficient approximately 11% lower than the control, confirming the effectiveness of this mix in enhancing long-term durability.

### 4.4. Comparison of Freeze–Thaw Resistance Under Curing Methods

**Figure 7:**
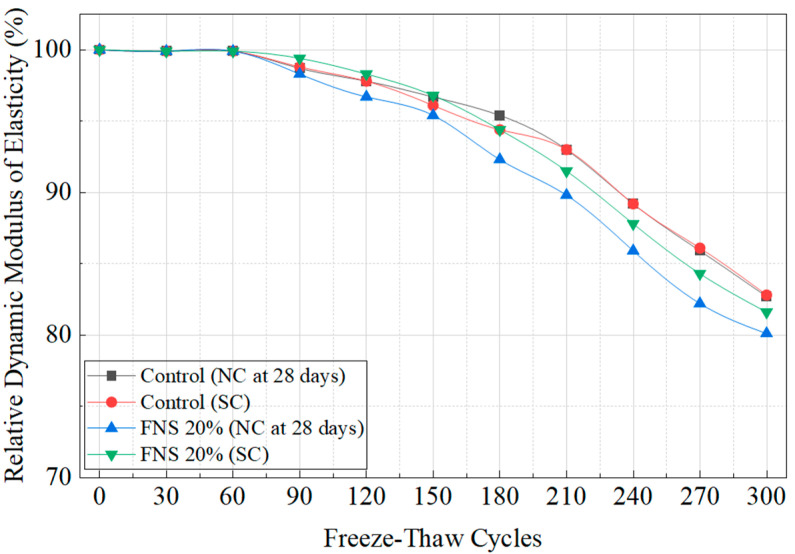
Freeze–thaw resistance of specimens under different curing methods at the optimal FNS replacement ratio.

The freeze–thaw resistance test was conducted according to ASTM C 666: Standard Test Method for Resistance of Concrete to Rapid Freezing and Thawing [33]. Detailed calculation formulas are provided in Appendix A.2. The control specimen and the optimal mix design incorporating FNS 20% were evaluated. During testing, the central temperature of the concrete specimens was maintained at −18 °C during freezing and +4 °C during thawing using a freeze–thaw chamber. Each cycle ensured that the maximum and minimum temperatures at the center of the specimen remained within the ranges of 4 °C and −18 °C, respectively. The temperature was strictly maintained to prevent the specimen from falling below −20 °C or exceeding +6 °C. Each freeze–thaw cycle consisted of 4 h of freezing followed by 4 h of thawing. The results are shown in Figure 7. After 300 cycles, the relative dynamic modulus of elasticity was 82.7% for the control specimen (NC, 28 days), 82.8% for the control specimen (SC), 80.1% for the FNS 20% specimen, and 81.6% for the FNS 20% specimen (SC). These results indicate that SC slightly improves freeze–thaw resistance compared to NC, with a more pronounced improvement in the FNS 20% specimen. Although the FNS 20% specimen exhibited slightly lower freeze–thaw resistance than the control, the difference was minimal. All specimens retained over 80% of their relative dynamic modulus of elasticity after 300 cycles, exceeding the 60% requirement of ASTM C666, which confirms adequate durability. In addition to compressive strength, split tensile strength, and chloride diffusion resistance, the FNS 20% mix also demonstrated balanced mechanical and durability performance in terms of freeze–thaw resistance. This mix can be considered optimal for precast concrete applications when combined with SC.

## 5. Conclusions

This study investigated the effects of partially replacing OPC with FNS on the performance of concrete, particularly under curing conditions representative of precast applications. A comprehensive evaluation, including heat of hydration, microstructural characteristics, mechanical properties, and durability indicators, was conducted. Based on the experimental findings, the following conclusions were drawn:

(1) All specimens reached peak adiabatic temperatures within 1–2 days, but higher FNS replacement ratios delayed hydration and early-age strength development. This effect is attributed to the elevated MgO content in FNS, as confirmed by SEM–EDS, which inhibits early hydration reactions and lowers heat release.
(2) XRD diffraction analysis showed that the CS-based phase and calcium hydroxide were generally higher under SC for the control, FNS 10%, and FNS 20%, indicating that SC can produce hydration effects comparable to 28-day NC, suggesting that SC can promote hydration and compensate for early-age strength reduction in FNS concrete, making it suitable for precast applications.
(3) Compressive and split tensile strength decreased with increasing FNS under NC. Under SC, FNS 10% achieved the highest strength, and FNS 20% performed similarly to the control. The results confirm that a 20% FNS replacement ratio provides the best balance between performance and sustainability, ensuring mechanical reliability while reducing clinker use.
(4) The FNS 20% revealed chloride penetration resistance (≈11% improvement under SC compared to NC) and adequate freeze–thaw durability, maintaining >80% of dynamic modulus after 300 cycles, surpassing the ASTM C666 requirement. Thus, FNS 20% combined with SC is considered an effective mix design for precast concrete applications.

## 6. Further Studies

Since the study has already been completed, quantitative data is not available at this time. Therefore, in future studies, more quantitative data from additional experiments, such as structural experiments, life cycle assessments, economic feasibility analyses, and the combined effect of durability, are needed for more precise application analysis.

## Figures and Tables

**Table 1:** CO_2_ emission reductions using different cement replacement materials.

Materials	Replacement Ratio (%)	CO_2_ Reduction Rate (%)	Reference Paper
GGBFS	70%	47	E. Crossin [7]
Silica fume	10–15	8–12	Aneel Kumar et al. [8]
Fly ash	25%	22–30	Orozco et al. [9]
FNS	25–45	77	Andres Arce et al. [10]

**Table 2:** Mix proportions of mortar with varying FNS replacement ratios.

Specimens	W	C	FNS	S	G	SP	W/B	Slump	Air Content	f_ck_
	kg/m^3^						%	mm	%	MPa
Control	175.00	437.50	-	700.10	978.00	1.60	40	100	5	35
FNS 10%		393.75	43.75							
FNS 20%		350.00	87.50							
FNS 30%		306.25	131.25							

**Table 3:** Comparison of the chemical composition between OPC and FNS.

	Element	CaO	SiO_2_	Al_2_O_3_	Fe_2_O_3_	SO_3_	MgO	K_2_O	Na_2_O	LOI
Specimen		Unit (%)								
OPC		60.99	20.47	4.95	3.27	2.53	3.14	1.07	0.33	2.43
FNS		6.60	40.70	2.70	7.90	0.70	43.40	-	-	0.01

**Table 4:** Oxide content per unit quantity for each specimen.

	Element	CaO	SiO_2_	Al_2_O_3_	Fe_2_O_3_	SO_3_	MgO	K_2_O	Na_2_O
Specimen		Unit (kg/m^3^)							
Control		223.3	74.95	18.12	11.97	9.26	11.50	3.92	1.21
FNS 10%		200.66	59.96	14.50	12.58	7.41	35.22	3.13	0.97
FNS 20%		183.49	89.77	16.48	15.36	7.92	40.98	3.13	0.97
FNS 30%		163.57	97.18	15.65	17.16	7.25	55.72	2.74	0.85

**Table 5:** Adiabatic temperature rise test results of Control and FNS.

Specimen	Time (Days)	Adiabatic Temperature (K)	Fastest Reaction (α)
Control	14	47.3	1.3220
FNS 10%		43.4	1.2050
FNS 20%		39.4	1.1370
FNS 30%		37.0	1.0980

**Table 6:** SEM-EDS results of specimens subjected to different curing methods.

	Specimens	wt% at NC (28 Days)			
Element		Control	FNS 10%	FNS 20%	FNS 30%
Ca		3.60	4.46	2.01	8.20
O		52.71	45.14	41.53	47.50
Si		16.05	10.09	14.23	14.89
Mg		0.59	2.38	13.13	14.44
Fe		3.64	10.52	8.48	4.16
	**Specimens**	**wt% at SC**			
**Element**		**Control**	**FNS 10%**	**FNS 20%**	**FNS 30%**
Ca		16.42	5.07	17.02	7.46
O		46.37	42.12	44.70	46.62
Si		7.39	8.34	6.75	15.82
Mg		1.45	2.60	2.45	9.13
Fe		0.97	7.30	2.33	3.95

**Table 7:** XRD intensities of specimens subjected to different curing methods.

NC (28 Days)				
Specimens	Ettringite	CS-Based Phase	C-F-S-H Phase	Calcium Hydroxide
	20.80°	26.60°	27.96°	27.42°
Control	9564	56,869	4647	4218
FNS 10%	10,925	56,860	7115	6930
FNS 20%	10,458	65,675	5255	3233
FNS 30%	10,274	73,276	3822	7760
**SC (28 Days)**				
**Specimens**	**Ettringite**	**CS-based phase**	**C-F-S-H phase**	**Calcium Hydroxide**
	**20.80°**	**26.60°**	**27.96°**	**27.42°**
Control	8144	57,503	3908	6025
FNS 10%	9410	58,053	3863	9232
FNS 20%	10,270	57,578	2169	4607
FNS 30%	9869	70,155	7602	4880

## Data Availability

The original contributions presented in the study are included in the article; further inquiries can be directed to the corresponding author.

## Appendix A

### Appendix A.1. Chloride Penetration Resistance Method

The appendix is a detailed durability section on the chloride migration coefficient. A 0.3 mol NaOH solution was used in the anode compartment, and a 10% NaCl solution was used in the cathode compartment. A constant voltage of 30 V was applied. To calculate the chloride diffusion coefficient, the initial current, temperature, and chloride penetration depth were measured using Equation (A1) [33]:

(A1) $$D_{nssm}=\frac{0.0239\left(273+T\right)L}{\left(U-2\right)t}(X_{d}-0.0238\sqrt{\frac{\left(273+T\right)LX_{d}}{U-2}})$$

where $D_{nssm}$ is the non-steady-state migration coefficient (m^2^/s), U is the applied voltage (V), and T is the average temperature of the anolyte solution (°C). L is the specimen thickness (mm), $X_{d}$ is the average chloride penetration depth (mm), and t is the test duration (h).

### Appendix A.2. Freeze–Thaw Resistance Method

The appendix is a detailed durability section on the freeze–thaw resistance. The relative dynamic modulus of elasticity was measured before and after 300 cycles for both the NC and SC specimens. It was calculated using Equations (A2) and (A3) as follows:

(A2) $$P_{c}=(\frac{{n_{c}}^{2}}{{n_{o}}^{2}})\times 100$$

where $P_{c}$ is the relative dynamic elasticity modulus (%) after cycle $N_{o}$, ${n_{c}}^{2}$ is the fundamental resonance frequency (Hz) after cycle $N_{o}$, and ${n_{o}}^{2}$ is the fundamental resonance frequency (Hz) before testing.

(A3) $$DF=\frac{P_{c}N_{o}}{M}$$

where DF is the durability factor, $P_{c}$ is the relative dynamic modulus of elasticity (%) at cycle $N_{o}$, $N_{o}$ is the number of cycles when $P_{c}\;$ reaches 60% or the number of cycles at the end of testing, M is the total number of freeze–thaw cycles.
